# Predictors of early growth in academic achievement: the head-toes-knees-shoulders task

**DOI:** 10.3389/fpsyg.2014.00599

**Published:** 2014-06-17

**Authors:** Megan M. McClelland, Claire E. Cameron, Robert Duncan, Ryan P. Bowles, Alan C. Acock, Alicia Miao, Megan E. Pratt

**Affiliations:** ^1^Human Development and Family Sciences, Oregon State UniversityCorvallis, OR, USA; ^2^Center for Advanced Study of Teaching and Learning, University of VirginiaCharlottesville, VA, USA; ^3^Department of Human Development and Family Studies, Michigan State UniversityEast Lansing, MI, USA

**Keywords:** executive function, self-regulation, academic achievement, early childhood, measurement

## Abstract

Children's behavioral self-regulation and executive function (EF; including attentional or cognitive flexibility, working memory, and inhibitory control) are strong predictors of academic achievement. The present study examined the psychometric properties of a measure of behavioral self-regulation called the Head-Toes-Knees-Shoulders (HTKS) by assessing construct validity, including relations to EF measures, and predictive validity to academic achievement growth between prekindergarten and kindergarten. In the fall and spring of prekindergarten and kindergarten, 208 children (51% enrolled in Head Start) were assessed on the HTKS, measures of cognitive flexibility, working memory (WM), and inhibitory control, and measures of emergent literacy, mathematics, and vocabulary. For construct validity, the HTKS was significantly related to cognitive flexibility, working memory, and inhibitory control in prekindergarten and kindergarten. For predictive validity in prekindergarten, a random effects model indicated that the HTKS significantly predicted growth in mathematics, whereas a cognitive flexibility task significantly predicted growth in mathematics and vocabulary. In kindergarten, the HTKS was the only measure to significantly predict growth in all academic outcomes. An alternative conservative analytical approach, a fixed effects analysis (FEA) model, also indicated that growth in both the HTKS and measures of EF significantly predicted growth in mathematics over four time points between prekindergarten and kindergarten. Results demonstrate that the HTKS involves cognitive flexibility, working memory, and inhibitory control, and is substantively implicated in early achievement, with the strongest relations found for growth in achievement during kindergarten and associations with emergent mathematics.

## Introduction

Self-regulation has been established as a key mechanism associated with a variety of outcomes including school readiness (Blair and Razza, 2007; McClelland et al., 2007a; Morrison et al., 2010), academic achievement during childhood and adolescence (McClelland et al., 2006; Cameron Ponitz et al., 2009; Duckworth et al., 2010; Li-Grining et al., 2010), and long-term health and educational outcomes (Moffitt et al., 2011; McClelland et al., 2013). Experts from diverse disciplines agree that self-regulation has important implications for individual health and well-being starting early in life (Geldhof et al., 2010; McClelland et al., 2010). Moreover, the behavioral aspects of self-regulation may be especially important for academic and school success (McClelland et al., 2007a; Cameron Ponitz et al., 2009; McClelland and Cameron, 2012). Given the multiple cognitive components involved in behavioral self-regulation, such as cognitive flexibility, working memory, and inhibitory control, measuring these skills during early childhood is challenging (Carlson, 2005; Cameron Ponitz et al., 2008; Caughy et al., 2014), and until recently, there have been few reliable and valid measures of these skills. Even fewer studies are able to address how well individual measures predict achievement growth over this significant developmental period or whether growth in behavioral measures are associated with growth in learning during the transition to kindergarten. The present study examined how a structured observation of behavioral self-regulation, the Head-Toes-Knees-Shoulders task (HTKS), was related to traditional executive function (EF) measures of cognitive flexibility, working memory, and inhibitory control. We also tested the predictive validity of these direct assessments for growth in academic achievement over four time points between preschool and kindergarten.

### Definitions of behavioral self-regulation and executive function

Children's self-regulation of their cognitions, emotions, and behavior is critical for their success throughout the school trajectory and in adulthood (Zelazo and Müller, 2002; Baumeister and Vohs, 2004; Blair and Razza, 2007; McClelland et al., 2007a, 2013; Rimm-Kaufman et al., 2009). Different disciplines have examined self-regulation and related constructs using a variety of terms. For example, scholars in the field of personality have used self-control to describe a set of skills similar to self-regulation and often refer to the integration of various self-control processes (Zimmerman, 2000; Eisenberg et al., 2014). And in the study of temperament, the construct of effortful control includes aspects of attentional focusing, inhibitory control, and regulating emotions, which are similar to self-regulation although temperament does not incorporate working memory (McClelland et al., 2010). In developmental psychology, self-regulation is a broad term that includes both top-down planning processes (e.g., executive functions or EF) and bottom-up regulation of more reactive impulses (Zelazo and Cunningham, 2007; Blair and Raver, 2012).

EF is a well-known construct originating in cognitive psychology that includes attentional or cognitive flexibility, working memory, and inhibitory control, which enables individuals to plan, organize, and problem-solve as well as to manage their impulses (Best and Miller, 2010). We have defined behavioral self-regulation as deliberately applying multiple component processes of attentional or cognitive flexibility, working memory, and inhibitory control to overt, socially contextualized behaviors like remembering to raise one's hand and waiting to be called upon instead of shouting out an answer (McClelland et al., 2007b; Cameron Ponitz et al., 2008; Morrison et al., 2010). Thus, whereas EF processes have typically been examined in terms of cognitive development, using materials and responses appropriate to the laboratory, behavioral self-regulation can be defined as the outward manifestation of those EF processes in adaptive, real-world behaviors (Cameron Ponitz et al., 2009; McClelland and Cameron, 2012). Throughout this paper we broadly refer to the set of contextualized, ecologically-relevant cognitive and behavioral processes as behavioral and use EF to refer specifically to individual cognitive components of attentional or cognitive flexibility, working memory, and inhibitory control. Whether a behavioral self-regulation measure is distinct from traditional EF measures in predicting academic achievement is one aim of this study.

The integration of EF into ecologically-relevant behaviors is critical for meeting school- and task-related demands and for successfully navigating early learning environments (McClelland and Cameron, 2012). For example, research indicates that behavioral self-regulation robustly contributes to achievement after controlling for initial achievement levels and other socio-demographic variables such as child IQ, age, ethnicity, and parent education level (Duncan et al., 2007; von Suchodoletz et al., 2009). In one recent study, a child with one standard deviation higher parent ratings of attention and persistence at age 4 had 49% higher odds of completing college by age 25 (McClelland et al., 2013). In another investigation, children with strong behavioral self-regulation in preschool had greater school age achievement after controlling for child IQ (von Suchodoletz et al., 2009).

The distinct roles played by the three individual EF components (attentional or cognitive flexibility, working memory, and inhibitory control) in regulating behavior is still debated (Barkley, 1997; Bronson, 2000; Müller et al., 2006). Attentional or cognitive flexibility allows children to shift focus and pay attention to new details, while simultaneously ignoring environmental distractions (Barkley, 1997; Rothbart and Posner, 2005). It may form the foundation for behavioral self-regulation and problem-solving (Zelazo and Müller, 2002; Rothbart and Posner, 2005; Rueda et al., 2005). Working memory allows children to remember and follow directions and helps them plan solutions to a problem (Gathercole and Pickering, 2000; Kail, 2003), and inhibitory control helps children stop one response in favor of a more adaptive behavior (Dowsett and Livesey, 2000; Carlson and Moses, 2001; Rennie et al., 2004).

Many measures of EF for young children produce a binary (pass/fail) distribution, which is consistent with Diamond et al. (2002) conceptualization of when children can keep track of multiple rules. In young children this depends on their ability to inhibit their initial impulse long enough to remember the rule and then give the correct response. Keeping track of and manipulating multiple rules (utilizing working memory) while also inhibiting initial impulses and activating an unnatural response is especially challenging for children. Our conceptualization of behavioral self-regulation is based on the notion that integrating aspects of EF allows children to control their behavior, remember instructions, pay attention, and complete learning tasks in school settings. In this study, we examined how well a measure of behavioral self-regulation tapped individual components of EF (cognitive flexibility, working memory, and inhibitory control) and how it predicted gains in academic achievement compared to these other EF measures.

### The HTKS measure of behavioral self-regulation

The HTKS measure of behavioral self-regulation integrates aspects of EF into a short game appropriate for children aged 4–8 years. Using no materials but rather relying on interactions between the examiner and the child, the HTKS has three sections with up to four paired behavioral rules: “touch your head” and “touch your toes;” “touch your shoulders” and “touch your knees.” Children first respond naturally, and then are instructed to switch rules by responding in the “opposite” way (e.g., touch their head when told to touch their toes). If children respond correctly after all four paired behavioral rules are introduced, the pairings are switched in the third section (i.e., head goes with knees and shoulders go with toes). In previous research (Cameron Ponitz et al., 2009; Wanless et al., 2011b; McClelland and Cameron, 2012), we have proposed that the HTKS measures behavioral self-regulation by requiring children to integrate into their behavior the following EF skills: (a) paying attention to the instructions, (b) using working memory to remember and execute new rules while processing the commands, (c) using inhibitory control through inhibiting their natural response to the test command while initiating the correct, unnatural response, and (d) using cognitive flexibility and working memory when rules accumulate and then change in the second and third sections.

Based on comparisons of HTKS scores to teacher ratings and parent reports of attention and inhibitory control, there is some evidence from previous research that the HTKS involves components of EF (McClelland et al., 2007a; Cameron Ponitz et al., 2009; Wanless et al., 2013). Other research has shown that the HTKS is significantly correlated with measures of working memory and requires children to successfully remember the changing rules of the task (Lan et al., 2011). However, some studies (including some of our own previous work, e.g., Fuhs and Day, 2011; Lan et al., 2011; Turner et al., 2012) describe the task as predominately tapping inhibitory control or response inhibition. Thus, it is unclear if the HTKS is best aligned with one of the individual EF components, or if there is empirical evidence for it as a separate measure of behavioral self-regulation requiring the integration of multiple components. This issue has not been directly examined using multiple direct assessments of cognitive flexibility, working memory, and inhibitory control. Thus, a goal of the present study was to examine how the HTKS related to direct assessments of EF in a sample of children aged 3–7 years.

### Predictors of academic achievement and school success

Children's developmental trajectories are shaped by dynamic and interacting factors such as maturation, early experience, and brain development, especially in the prefrontal cortex (Diamond, 2002; Blair and Diamond, 2008; Blair and Raver, 2012). These factors also make the early childhood years a sensitive period for the development of behavioral self-regulation. Thus, given the potential malleability of behavioral self-regulation and related EF components, the early childhood years are an especially important time to examine relations between behavioral self-regulation and early academic achievement.

Of particular interest in the current study is the notion that behavioral self-regulation and EF processes are foundational for learning in a variety of domains especially in early childhood classrooms. Further, the pattern of skills that most strongly contributes to concurrent achievement may differ from skills that are important later in a child's developmental trajectory (Paris, 2005; Murrah, 2010). With regard to EF components, the development of inhibitory control is thought to occur first making it possible for children to demonstrate cognitive flexibility (Diamond et al., 2002; Best and Miller, 2010). These processes develop alongside working memory, though the development of this component is relatively more protracted with maturational improvements documented through adolescence (Best and Miller, 2010). One question these findings raise is which EF component(s) contribute the most to behavioral self-regulation at different ages across the early childhood span (and whether the components are the same or different across the prekindergarten and kindergarten years). In addition, the question of what skills and measures are most strongly associated with academic learning over the transition to school becomes important to address. This study examined the predictive validity of a measure of behavioral self-regulation and three EF component tasks to growth in academic achievement. We used random effect models and fixed effects models to examine predictive relations of each task to academic outcomes during the preschool and kindergarten years.

### Testing the strength of the association between behavioral self-regulation and academic outcomes

A number of recent studies have examined the strength of associations between behavioral self-regulation and academic outcomes concurrently and longitudinally (Welsh et al., 2010; McClelland et al., 2013; Weiland and Yoshikawa, 2013). There is consistently strong evidence that behavioral self-regulation and EF significantly predict academic outcomes, even after controlling for baseline achievement levels, child IQ, and a host of demographic variables (e.g., McClelland et al., 2006, 2007a, 2013; Blair and Razza, 2007; Welsh et al., 2010; Moffitt et al., 2011). Relations have been especially strong for behavioral self-regulation and EF skills predicting growth in children's mathematics achievement (Blair and Razza, 2007; Cameron Ponitz et al., 2009; Bull et al., 2011).

Previous research on the relation between behavioral self-regulation, EF, and growth in academic outcomes has almost always utilized a *random effects approach (REA)*, in which the child is treated as a random draw from a distribution of individual differences in the rate of growth in academic skills. Such an approach can lead to biased estimates of how strongly a variable predicts growth when there are other time-invariant predictors of growth not included in the model (Clark and Linzer, 2012). An alternative approach, a *fixed effects approach (FEA)*, instead treats each child as a fixed effect (Allison, 2009), which eliminates this source of bias but at the expense of adding a large number of parameters associated with each child. The additional parameters (i.e., the fixed effect of each child in this case) mean the FEA can have lower power than the REA. To summarize, the REA can be used to examine inter-individual differences on behavioral self-regulation and explain these differences while modeling measured covariates that could be associated with behavioral and academic achievement (i.e., child IQ, age, parental education). The FEA can be used to investigate the association between intra-individual change over time in a child's behavioral self-regulation or EF skills and academic achievement.

In a study of 3- to 6-year-old children (*N* = 794), Willoughby and colleagues found that significant predictive relations between EF and academic achievement using a random effects approach became non-significant when using FEA (Willoughby et al., 2012b). Based on these results, Willoughby et al. (2012b) argued that the widely reported associations between EF and achievement might be spurious and driven by unmeasured time-invariant characteristics of the child. This argument, however, should be evaluated with caution. First, the null result could be attributable to a lack of power for a FEA to detect substantively significant effects rather than actual null effects. Second, the Willoughby et al. (2012b) study included just two time points (with an average of 4.4 months between time one and time two), so development in academic achievement may not have progressed sufficiently for individual differences in change to manifest. Furthermore, only two measures of EF (balance beam and pencil tapping) were used. Thus, it may not be surprising that there was no significant relation between the EF components that were measured and academic achievement in this study.

In addition, FEA findings tend to be sample specific (Allison, 2009; Clark and Linzer, 2012) making it difficult to generalize beyond any given study. This is partly because the sensitivity of a measure to change also depends on the validity and variability of the measure over time. This makes it important to replicate findings using different samples of children, with multiple measures and multiple time points. The current study sought to further test the strength of associations between behavioral self-regulation and academic achievement in young children using multiple measures of EF and behavioral self-regulation over the early school transition. Specifically, using both FEA and REA, we explored to what extent four measures of EF and the HTKS measure of behavioral self-regulation significantly predicted achievement growth over four waves of data from the fall of prekindergarten to the spring of kindergarten. We anticipated that the two models would demonstrate the same overall pattern of results, especially for children's early mathematics skills. We anticipated that these results would be consistent across the two analytical approaches because we include more occasions of measurement and more measures of EF than the previous study using the lower powered FEA (Willoughby et al., 2012b).

### The present study

The present study examined the longitudinal and psychometric properties of the HTKS measure of behavioral self-regulation by assessing: (1) construct validity through relations with traditional EF tasks, and (2) predictive validity for emergent literacy, vocabulary, and mathematics skills using random effects and fixed effects models. First, we anticipated that the HTKS would significantly relate to measures of cognitive flexibility, working memory, and inhibitory control based on previous research (McClelland et al., 2007a,b; Cameron Ponitz et al., 2009; Lan et al., 2011). Second, we considered predictive validity using random effects and fixed effects models between prekindergarten and kindergarten (over 4 time points). Based on previous research (e.g., Cameron Ponitz et al., 2009), we expected that compared to individual measures of cognitive flexibility, working memory, and inhibitory control, the HTKS would emerge as the strongest predictor of growth in academic achievement (literacy, vocabulary, and mathematics) in kindergarten. We also expected that the HTKS and measures of EF would be especially predictive of growth in early mathematics skills (Bull and Scerif, 2001; Cameron Ponitz et al., 2009; Bull et al., 2011).

## Method

### Participants and procedure

The sample included 208 children (50% male) who participated in at least one wave of data collection (see Table 1). Families were recruited from 28 classrooms and 16 preschools located in the Pacific Northwest United States. The following kindergarten year, children were in 63 classrooms and 33 schools. Of the 208 children, 204 participated during wave 1; four children were not tested during wave 1 because they either refused testing sessions (*n* = 3) or parents asked for their child to be included during later waves (*n* = 1; see Table 1 for total sample size by assessment and wave). Children and families were recruited through letters in an enrollment packet sent during the summer prior to the preschool year. Consent was obtained from a parent of all children in the study, and families were given $20 gift cards at each time point of the study.

**Table 1:** **Descriptive statistics of covariates, the HTKS and other EF measures, and achievement outcomes across four waves**.

	**Fall prekindergarten**		**Spring prekindergarten**		**Fall kindergarten**		**Spring kindergarten**	
	***N***	***M* (*SD*)**	***N***	***M* (*SD*)**	***N***	***M* (*SD*)**	***N***	***M* (*SD*)**
Age	204	55.67 (4.42)	197	61.27 (4.45)	157	67.97 (3.88)	154	73.89 (3.87)
Percent male	204	50%	197	51%	157	54%	154	55%
Percent head start	204	50%	197	50%	157	45%^h^	154	47%^h^
Percent ELL^a^	204	14%	197	14%	157	13%	154	12%
Parent education	179	14.80 (3.68)	175	14.69 (3.67)	144	15.06 (3.76)	142	15.12 (3.76)
HTKS^b^	198	17.38 (16.92)	196	24.73 (18.61)	153	34.30 (17.60)	152	40.19 (15.27)
DCCS^c^	202	13.35 (6.72)	194	16.29 (6.09)	157	19.11 (4.49)	151	19.99 (3.47)
Day-Night Stroop	198	23.50 (9.12)	193	26.31 (7.90)	156	29.22 (4.29)	152	29.55 (3.95)
Working memory^d^	198	449.65 (15.10)	192	457.30 (18.69)	153	464.84 (19.75)	150	474.68 (19.29)
Simon Says	200	0.70 (1.38)	190	1.28 (1.92)	156	2.28 (1.93)	149	2.83 (1.84)
Mathematics^e^	197	409.31 (25.50)	194	419.99 (22.75)	155	434.14 (18.85)	152	444.39 (17.08)
Early literacy^f^	200	338.24 (25.65)	194	352.32 (26.45)	155	372.34 (29.46)	151	405.66 (36.93)
Vocabulary^g^	201	468.11 (14.06)	195	473.00 (11.72)	155	476.86 (12.21)	149	478.57 (11.34)

a ELL = English Language Learner Status.

b The Head-Toes-Knees-Shoulders task.

c The Dimensional Change Card Sort task.

d The Woodcock-Johnson Auditory Working Memory subtest.

e The Woodcock-Johnson Applied Problems subtest.

f The Woodcock-Johnson Letter-Word Identification subtest.

g The Woodcock-Johnson Picture Vocabulary Subtest.

h Percent in Head Start is based on the child's prekindergarten year.

Children were followed between preschool and kindergarten, with assessments in the fall and spring of each year (4 waves total). Children were assessed in English or Spanish in 2–3 sessions lasting 10–15 min each. About 50% of the children were enrolled in Head Start during the preschool year. At fall of preschool, children ranged in age from 36- to 65-months old (*M* = 55.67, *SD* = 4.42). Parent education ranged from about 5–23 years, with an average of approximately 3 years of college (*M* = 14.80, *SD* = 3.68 at baseline). Children were 61% White; 18% Latino; 0.5% African American; 1% Middle Eastern; 13% multiracial; and 1% other. About 14% of the sample was Spanish-speaking and were assessed in Spanish. In this sample, all Spanish-speaking children were identified as low-income. Moreover, low-income Spanish-speaking families reported significantly lower parent education levels, [*t*_(85)_ = 4.958, *p* < 0.001], such that the parents of children who were Spanish-speaking reported lower levels of education (*M* = 10.10 years) than low-income English speakers (*M* = 12.66 years). In addition, compared to their low-income English-speaking peers, in the fall of preschool, Spanish-speaking children from low-income families scored significantly lower on the HTKS, [*t*_(95)_ = 2.83, *p* = 0.006], some measures of EF [Dimensional Change Card Sort (DCCS), *t*_(99)_ = 2.14, *p* = 0.035, and Woodcock-Johnson Auditory Working Memory (WJ-WM) *t*_(98)_ = 3.77, *p* < 0.001], math, [*t*_(97)_ = 4.41, *p* < 0.001], and literacy, [*t*_(97)_ = 3.90, *p* < 0.001], but scored significantly higher on vocabulary scores, [*t*_(98)_ = −2.51, *p* = 0.014].

Current research has focused on including diverse samples of children to appropriately assess EF in different populations. We included both Spanish-speaking and English-speaking children to examine our research questions in diverse groups. Previous research with different samples of low-income children who were Spanish-speaking or English-speaking did not find significant differences at the fall of prekindergarten in children's HTKS or EF scores (e.g., Wanless et al., 2011b; Schmitt et al., under review). Thus, we included both groups of children based on previous work evaluating the two groups separately.

### Measures

#### Measures of behavioral self-regulation and EF

Children were assessed in preschool and kindergarten on the HTKS, Three-Dimensional Change Card Sort (DCCS), Day-Night Stroop task, the Auditory Working Memory subtest from the Woodcock-Johnson III Tests of Cognitive Abilities, and the Simon Says task. All tasks were counterbalanced to avoid order effects.

***HTKS***. The HTKS was used to assess children's behavioral self-regulation and requires cognitive flexibility, working memory, and inhibitory control (McClelland and Cameron, 2012). There are a total of 30 test items with scores of 0(*incorrect*), 1(*self-correct*), or 2(*correct*) for each item. A self-correct is defined as any motion to the incorrect response, but self-correcting and ending with the correct action. Scores range from 0 to 60 where higher scores indicate higher levels of behavioral self-regulation. The task takes approximately 5–7 min with strong inter-rater reliability (κ = 0.90; Cameron Ponitz et al., 2009; McClelland and Cameron, 2012). There are two parallel forms of the HTKS: A and B, which were given randomly in an alternating order of assessments over the four time points of the longitudinal study. Form A starts with head/toes and Form B starts with knees/shoulders. No significant differences have been found between the two versions of the task McClelland et al., 2007a; Cameron Ponitz et al., 2009; Wanless et al., 2011a; Bowles et al., submitted. The measure now incorporates three sections, the HTT (1 section of “opposites”), the HTKS (2 sections, two sets of “opposites”) and the HTKS—Extended (3 sections, adding a final rule switch). The task is available in a number of languages, is reliable, and significantly predicts academic outcomes in diverse samples (McClelland et al., 2007a,b; Wanless et al., 2011a; McClelland and Cameron, 2012; von Suchodoletz et al., 2013). Validity information for the current sample is presented in the Results below. Cronbach's alphas were computed in Mplus 7 using polychoric correlations, which are appropriate for categorical data. The HTKS in the current sample had Cronbach's alphas of 0.92, 0.94, 0.94, and 0.94 across the four waves of the study.

To assess inter-rater reliability in the current study, a random subsample of children (*n* = 28) was videotaped while being administered the HTKS task. Videotapes were later viewed and scored by an assessor who had not administered the original HTKS task to the child. We used double-coded HTKS sum scores analyzed with the default weighted kappa option in Stata (i.e., 1.00, 0.50, 0.00). The correlation between the double-coded HTKS scores was strong (*r* = 0.88, *p* < 0.001). Results showed high inter-rater agreement (92.29%), with a weighted Cohen's kappa of 0.79 (*p* < 0.001) indicating very strong inter-rater reliability for the HTKS task (Landis and Koch, 1977). To measure test-retest stability of the HTKS task in the current sample, Pearson's correlation coefficients for fall and spring HTKS scores were examined in prekindergarten and kindergarten (see Table 2). The average length of time between fall and spring HTKS task assessments was 5.64 months in prekindergarten (*SD* = 0.57, range = 4.17–7.16) and 5.84 months in kindergarten (*SD* = 0.81, range = 3.38–7.46). Results showed good test-retest stability with strong positive correlations between fall and spring HTKS total scores in both prekindergarten (*r* = 0.60, *p* < 0.001) and kindergarten (*r* = 0.74, *p* < 0.001).

**Table 2:** **Correlations of HTKS with other measures of EF during prekindergarten (*N* = 185–198) and kindergarten (*N* = 146–156)**.

**Variable**	**1**	**2**	**3**	**4**	**5**	**6**	**7**	**8**	**9**	**10**
1. Fall HTKS^a^	–	0.53^***^	0.29^***^	0.47^***^	0.44^***^	0.74^***^	0.33^***^	0.37^***^	0.59^***^	0.50^***^
2. Fall DCCS^b^	0.56^***^	–	0.23^***^	0.31^***^	0.41^***^	0.46^***^	0.51^***^	0.18^*^	0.41^***^	0.43^***^
3. Fall Day-Night Stroop	0.40^***^	0.36^***^	–	0.21^**^	0.13	0.22^**^	0.28^***^	0.63^***^	0.28^***^	0.12
4. Fall working memory^c^	0.41^***^	0.28^***^	0.20^**^	–	0.36^***^	0.38^***^	0.24^**^	0.19^*^	0.53^***^	0.25^**^
5. Fall Simon Says	0.38^***^	0.36^***^	0.31^***^	0.32^***^	–	0.42^***^	0.32^***^	0.19^*^	0.41^***^	0.58^***^
6. Spring HTKS^a^	0.60^***^	0.54^***^	0.34^***^	0.45^***^	0.32^***^	–	0.37^***^	0.27^***^	0.60^***^	0.48^***^
7. Spring DCCS^b^	0.46^***^	0.63^***^	0.31^***^	0.26^***^	0.28^***^	0.54^***^	–	0.25^**^	0.31^***^	0.36^***^
8. Spring Day-Night Stroop	0.31^***^	0.27^***^	0.41^***^	0.17^*^	0.13^†^	0.37^***^	0.32^***^	–	0.20^*^	0.24^**^
9. Spring working memory^c^	0.39^***^	0.40^***^	0.29^***^	0.38^***^	0.30^***^	0.39^***^	0.35^***^	0.26^***^	–	0.43^***^
10. Spring Simon Says	0.39^***^	0.47^***^	0.33^***^	0.40^***^	0.52^***^	0.54^***^	0.32^***^	0.21^**^	0.39^***^	–

Correlations on the bottom diagonal are for prekindergarten. Correlations on the top diagonal are for kindergarten.

a The Head-Toes-Knees-Shoulders task.

b The Dimensional Change Card Sort task.

c The Woodcock-Johnson Auditory Working Memory subtest.

† p < 0.10;

* p < 0.05;

** p < 0.01;

*** p < 0.001.

***Dimensional Change Card Sort (DCCS)***. Cognitive flexibility was assessed in English or Spanish using an adapted version of the Dimensional Change Card Sort (Deák, 2003; Hongwanishkul et al., 2005; Zelazo, 2006; Cepeda and Munakata, 2007), which is reliable and valid for children ages 3–5 years. Children were presented with cards that differed based on shape (i.e., dog, fish, bird), color (i.e., red, yellow, blue), and size (small, medium, large), and they were instructed to sort cards by each of the three dimensions. Children are first given six trials to sort by shape, then six trials to sort by color, then six trials to sort by size. If children scored at least five points on the sorting by size trial, children are given six more trials where they sorted cards by color and size depending on a border rule. The score is the sum of the total number of cards correctly sorted (1 = correct, 0 = incorrect) and scores can range from 0 to 24. In the current sample, the DCCS (using tetrachoric correlations) had Cronbach's alphas of 0.90, 0.92, 0.93, and 0.93 across four study waves.

***Auditory working memory***. The Auditory Working Memory test from the Woodcock-Johnson III Tests of Cognitive Abilities (Woodcock et al., 2001b) or The Bateria III Woodcock- Muñoz (Muñoz-Sandoval et al., 2005b) was used to assess children's working memory, the ability to remember and cognitively manipulate information. It demonstrates strong internal reliability: 0.93–0.96 for English-speaking preschool children and 0.77–0.79 for Spanish-speaking children. Cronbach's alphas are not available for the current sample because scores were entered at the subtest level; however, it has a reported strong median split-half reliability of 0.93 for children 4–7 years old (Mather and Woodcock, 2001).

***Day-Night Stroop task***. Inhibitory control was assessed using the Day-Night Stroop task in English or Spanish (Gerstadt et al., 1994; Berwid et al., 2005). Children are shown a series of 16 cards with pictures of a sun or moon and asked to say the opposite of what they see, saying “day” for a moon and “night” for a sun. Each of the 16 items were coded as 0 for an incorrect response, 1 for a self-correct or similar (i.e., saying “sun” when the correct response is “day”) response, or 2 for a correct response, with a possible range of 0–32. In the current sample, the Day-Night Stroop had Cronbach's alphas (using polychoric correlations) of 0.99, 0.99, 0.95, and 0.93 across four study waves.

***Simon Says task***. Inhibitory control was also assessed using Simon Says in English or Spanish. The measure is appropriate for prekindergarten and kindergarten children and has shown strong reliability and validity (Strommen, 1973; Carlson, 2005). Children are asked to perform an action only if the experimenter said “Simon says,” but to remain still otherwise. Thus, the task measures inhibition but not inhibition plus activation, which is required for the HTKS. Of the 10 total trials, the 5 trials requiring inhibition are scored (0 = incorrect/imitation 1 = correct/anti-imitation) and children are given a proportion score of the number correct (anti-imitation) on these 5 trials. In the current sample, task scores ranged from 0 to 5 and had Cronbach's alphas (using tetrachoric correlations) of 0.95, 0.98, 0.93, and 0.91 across four waves.

We chose two measures of inhibitory control because we wanted to differentiate responses requiring inhibition only (children must stop or control motor activity), as in Simon Says, from those requiring inhibition of a dominant response plus activation of another, non-dominant response, as in Day-Night (Kochanska et al., 1996; Blair, 2003). This enabled us to examine which type of inhibition contributes the most to HTKS performance.

#### Academic achievement outcomes

Children's early reading, vocabulary, and math skills were assessed on the Woodcock Johnson Psycho-Educational Battery-III Tests of Achievement (WJ-III; Woodcock et al., 2001a) in English or the Batería III Woodcock-Muñoz (Muñoz-Sandoval et al., 2005a) in Spanish. Large-scale studies using item-response theory (IRT) have equated the English and Spanish WJ measures and indicate that they assess the same competencies (Woodcock and Muñoz-Sandoval, 1993). Recent research indicates no significant differences on scores between the English and Spanish versions of the WJ-III (Hindman et al., 2010).

***Letter-word identification***. Children's early literacy skills were measured using the Letter-Word Identification subtest of the WJ-III (Woodcock et al., 2001a) or The Bateria III Woodcock-Muñoz (Muñoz-Sandoval et al., 2005a). This test measures letter skills and developing word-decoding skills. Published split-half reliabilities for English-speaking preschool and kindergarten children range between 0.98–0.99 and 0.84–0.98 for Spanish-speaking children. The Letter-Word Identification subtest has a median split-half reliability of 0.98 for children 4–7 years old (Mather and Woodcock, 2001).

***Picture vocabulary***. Children's receptive and expressive vocabulary skills were assessed with the Picture Vocabulary subtest of the WJ-III or The Bateria III Woodcock-Muñoz. Published split-half reliabilities for English-speaking children range between 0.76–0.81 and 0.88–0.89 for Spanish-speaking children. The Picture Vocabulary subtest has a median split-half reliability of 0.73 for children 4–7 years old (McGrew and Woodcock, 2001).

***Applied problems***. The Applied Problems subtest of the WJ-III or The Bateria III Woodcock-Muñoz was used to assess children's early mathematical operations needed to solve practical problems. Published split-half reliabilities for 4- and 5-year-old English-speaking children are 0.92–0.94 and 0.93–0.95 for Spanish-speaking children. The Applied Problems subtest has a median split-half reliability of 0.92 for children 4–7 years old (McGrew and Woodcock, 2001).

#### Parent demographic questionnaires

All parents completed a demographic questionnaire including background characteristics such as child age, English Language Learner status, parent education level, and gender. These variables were used as covariates.

## Results

### Analytic strategy

All research questions were addressed using Stata 13.1 (StataCorp, 2013). For construct validity, we first analyzed correlations between the HTKS and the four EF measures (the Day-Night Stroop, the DCCS, Simon Says, and the Woodcock-Johnson Working Memory subtest) for each wave. Then, we looked at multilevel models predicting HTKS scores with the four EF measures at each wave, controlling for child age, parent education, gender, Head Start status, and English Language Learner status. The *ICC*s for the HTKS across the four waves of data were: 0.12, 0.22, 0.15, and 0.10.

For predictive validity, we used multilevel models with generalized structural equation modeling in Stata 13.1, adjusting for the nested nature of the data (children within classrooms) and used a full information maximum likelihood estimator. For each random effects model, the models incorporated two waves of data, roughly 6 months apart during the same academic year (e.g., prekindergarten or kindergarten). In these models, the spring achievement variable was regressed on fall achievement, a single EF measure of interest, child age, parent education, gender, Head Start status, and English Language Learner status. The *ICC*s for the outcome achievement measures in the spring of prekindergarten (*ICC*s = 0.14–0.23) and kindergarten (*ICC*s = 0.22–0.27) suggested multilevel models were appropriate, and thus, all predictive models adjusted for this nesting.

Fixed effects analyses were estimated in Stata 13.1, with standard errors adjusted for clustering. In the fixed effects analyses, all four waves of data were analyzed simultaneously, such that all available data for each child from fall of prekindergarten to spring of kindergarten was modeled. In fixed effects analyses, associations of intra-individual change on predictors (i.e., EF) and outcomes (i.e., achievement) are of interest, thus no time-invariant covariates are included (as they were in the random effects model). Other than the effect of time, no time-varying covariates were used in these models (all time-invariant variables, measured and unmeasured, are incorporated in the estimate of the unit on the outcome).

#### Missing data, attrition, and descriptive statistics

Overall, there was relatively little missing data other than data lost due to attrition between the spring of prekindergarten and the fall of kindergarten (Waves 2–3). In the fall of prekindergarten (Wave 1), 204 children participated in the study. The most missing data on any assessment during the first wave occurred for the WJ-III Applied Problems subtest (*N* = 197) with 3.43% missing. In the spring of prekindergarten (Wave 2), a total of 197 children participated (97% retention from Wave 1 participants). The Simon Says task showed the most missing data with 3.55% missing.

In the fall of kindergarten (Wave 3, *N* = 157), 20.30% of the sample was lost due to attrition. Three covariates significantly predicted attrition from spring of prekindergarten to fall of kindergarten (year 1–2). Children were less likely to remain in the study if they were enrolled in Head Start during year 1, had parents with lower reported education levels, and were younger in age. Although differential attrition can lead to bias in parameter estimates, the use of covariates that predicted attrition (i.e., Head Start status, parental education, and age) with full information likelihood estimators are shown to provide reliable parameter estimates (Steiner et al., 2010).

In the fall of kindergarten (Wave 3), the task with the most missing was the HTKS with 2.55% missing data. From fall of kindergarten to spring of kindergarten (Wave 4, *N* = 154) there was a 98.09% retention rate. Of the participating children in Wave 4, the WJ-III Picture Vocabulary subtest and the Simon Says task showed the most missing with 3.25% missing data.

Descriptive statistics for covariates included in the models, parent-reported educational attainment, EF tasks, and achievement tasks are provided in Table 1. Furthermore, mean child performance improved in each EF measure and achievement measure across each wave of the study. In prekindergarten, children were clustered in 28 different classrooms (*M* = 7.42, range = 1–14), and by kindergarten, they had dispersed and were clustered in 63 different classrooms (*M* = 2.50, range = 1–10). We used full information maximum likelihood (FIML) to account for the small amount of missing data (Acock, 2012).

***RQ 1: construct validity of the HTKS***. Relations between the HTKS and each of the direct EF assessments of cognitive flexibility (DCCS), working memory (WJ-III Working Memory subtest), and inhibitory control (Day-Night, Simon Says) are presented for fall and spring of prekindergarten and kindergarten, with all correlations significant at = 0.001 (see Table 2). Overall, the HTKS was moderately correlated with the four direct assessments of EF throughout the four waves of data, suggesting convergent validity with traditional assessments of EF and construct validity that the HTKS assesses cognitive flexibility, working memory, and inhibitory control. For the fall of prekindergarten, the HTKS correlations with other EF tasks ranged from *r*s = 0.38–0.56 and for the spring of prekindergarten, correlations with other EF tasks ranged from *r*s = 0.37–0.54. For the fall of kindergarten, the HTKS correlations with other EF tasks ranged from *r*s = 0.29–0.53, and for the spring of kindergarten, correlations with other EF tasks ranged from *r*s = 0.27–0.60. Between prekindergarten and kindergarten, correlations among the EF measures ranged from *r*s = 0.20–0.56. The correlation between the HTKS and the DCCS was the strongest for the first three waves of data (*r*s from 0.46 to 0.56); however, by the spring of kindergarten (wave four) the HTKS was slightly more related to the measure of working memory (*r* = 0.60; see Table 2).

After examining correlations, we used multilevel models treating the HTKS as an outcome predicted concurrently by the four EF measures and controlling for child age, parent education, gender, Head Start status, and English Language Learner status (see Table 3). Results were similar to the correlational findings but also revealed that (1) EF measures were independently related to the HTKS and (2) relative relations differed by wave. In the fall of prekindergarten, all four tasks significantly predicted the HTKS measure with the cognitive flexibility task (DCCS) having the relatively largest effect (β = 0.36, *p* < 0.001). In the spring of prekindergarten, the Simon Says inhibitory control task was the most predictive of HTKS scores (β = 0.32, *p* < 0.001), with only working memory being non-significant. In the fall of kindergarten, by contrast, the DCCS and working memory were the only significant predictors of the HTKS, with the DCCS having the largest effect (β = 0.28, *p* < 0.001). In the spring of kindergarten, the working memory and the Simon Says tasks were the only significant predictors, with working memory having the largest relative effect (β = 0.42, *p* < 0.001) on HTKS scores.

**Table 3:** **Construct validity: multilevel regressions of EF measures predicting HTKS during prekindergarten (*N* = 196–198) and Kindergarten (*N* = 152–153)**.

**Predictor**	**Prekindergarten**						**Kindergarten**					
	**Fall HTKS^a^**			**Spring HTKS^a^**			**Fall HTKS^a^**			**Spring HTKS^a^**		
	***B***	***SE***	**β**	***B***	***SE***	**β**	***B***	**SE**	**β**	***B***	***SE***	**β**
DCCS^b^	0.90	0.18	0.36^***^	0.72	0.18	0.24^***^	1.12	0.29	0.28^***^	0.42	0.30	0.10
Day-Night Stroop	0.27	0.13	0.14^*^	0.36	0.14	0.15^*^	0.41	0.26	0.10	0.13	0.25	0.03
Working memory^c^	0.21	0.07	0.19^**^	0.04	0.06	0.04	0.17	0.07	0.19^*^	0.34	0.06	0.42^***^
Simon Says	1.81	0.80	0.15^*^	3.12	0.57	0.32^***^	1.05	0.69	0.12	1.83	0.61	0.22^**^

Covariates (not shown) include parental education, child age (in months), Head Start status, gender, and English Language Learner status.

a The Head-Toes-Knees-Shoulders task.

b The Dimensional Change Card Sort task.

c The Woodcock-Johnson Auditory Working Memory subtest.

* p < 0.05;

** p < 0.01;

*** p < 0.001.

***RQ 2: predictive validity of the HTKS and EF measures to academic outcomes***. Random effects multilevel models were used to examine inter-individual differences on behavioral self-regulation and EF predicting improvement on achievement measures in each academic year (predictive validity). Results of multilevel regressions (i.e., predicting spring achievement from fall EF during the same academic year while controlling for fall achievement) indicated that Wave 1 prekindergarten performance on the HTKS, DCCS (cognitive flexibility), and Day-Night Stroop (inhibitory control) tasks predicted Wave 1-Wave 2 improvement in early mathematics (β = 0.14, *p* = 0.007; β = 0.17, *p* = 0.002; β = 0.14, *p* = 0.006, respectively; see Table 4). The DCCS and working memory tasks also predicted improvement in early vocabulary (β = 0.11, *p* = 0.040; β = 0.10, *p* = 0.020, respectively). None of the fall tasks significantly predicted early literacy improvement during the prekindergarten year.

**Table 4:** **Predictive validity: random effects models for the HTKS and other EF measures predicting achievement growth in prekindergarten (*N* = 194–195) and kindergarten (*N* = 149–152)**.

**Predictor**	**Mathematics^d^**			**Early literacy^e^**			**Vocabulary^f^**		
	***B***	***SE***	**β**	***B***	***SE***	**β**	***B***	***SE***	**β**
**PREKINDERGARTEN**									
HTKS^a^	0.19	0.07	0.14^**^	0.07	0.08	0.04	0.04	0.03	0.06
DCCS^b^	0.59	0.19	0.17^**^	0.23	0.21	0.06	0.20	0.10	0.11^*^
Day-Night Stroop	0.36	0.13	0.14^**^	0.09	0.15	0.03	0.09	0.07	0.07
Working memory^c^	0.05	0.07	0.03	0.09	0.08	0.05	0.08	0.03	0.10^*^
Simon Says	1.08	0.77	0.07	1.18	0.86	0.06	0.56	0.39	0.07
**KINDERGARTEN**									
HTKS^a^	0.15	0.06	0.15^*^	0.36	0.11	0.17^**^	0.10	0.04	0.16^**^
DCCS^b^	0.25	0.22	0.07	0.57	0.41	0.07	0.22	0.13	0.09^†^
Day-Night Stroop	0.01	0.19	0.00	0.50	0.40	0.06	0.27	0.12	0.10^*^
Working memory^c^	0.15	0.05	0.17^**^	0.14	0.10	0.07	0.02	0.03	0.04
Simon Says	1.03	0.50	0.12^*^	0.37	1.01	0.02	0.82	0.32	0.14^*^

Covariates (not shown) include parental education, child age (in months), Head Start status, gender, and English Language Learner status. Spring achievement gains control for fall achievement. Full Information Maximum Likelihood (FIML) estimation used to deal with missing data.

a The Head-Toes-Knees-Shoulders task.

b The Dimensional Change Card Sort task.

c The Woodcock-Johnson Auditory Working Memory subtest.

d The Woodcock-Johnson Applied Problems Subtest.

e The Woodcock-Johnson Letter-Word Identification subtest.

f The Woodcock-Johnson Picture Vocabulary Subtest.

† p < 0.10;

* p < 0.05;

** p < 0.01.

Over the kindergarten year, Wave 3 scores on the HTKS, working memory, and Simon Says tasks predicted improvement in early mathematics (β = 0.15, *p* = 0.018; β = 0.17, *p* = 0.002; β = 0.12, *p* = 0.038, respectively; see Table 4). The HTKS task was the only task to significantly predict early literacy improvement (β = 0.17, *p* = 0.001). The HTKS, the Day-Night Stroop, and the Simon Says tasks significantly predicted kindergarten vocabulary improvement (β = 0.16, *p* = 0.003; β = 0.10, *p* = 0.023; β = 0.14, *p* = 0.011, respectively), with trend level effects on vocabulary for the DCCS (β = 0.09, *p* = 0.095).

Fixed effects models were run next to examine intra-individual change in behavioral self-regulation and EF predicting intra-individual change in the academic outcomes over the four time points. Results generally matched the findings of the random effects models, with some weaker associations: growth in the HTKS, the DCCS, and the Day-Night Stroop all significantly predicted growth in mathematics (β = 0.10, *p* = 0.003; β = 0.09, *p* = 0.001; β = 0.07, *p* = 0.007; respectively; see Table 5). For example, for each standard deviation increase on the HTKS, children made a 2.5 point gain on math. Thus, children who showed the most growth in behavioral self-regulation and EF also demonstrated the most growth in mathematics between prekindergarten and kindergarten. In addition, the Day-Night Stroop was the only task that significantly predicted improvement in vocabulary development (β = 0.06, *p* = 0.039). Thus, children making improvements in inhibitory control, as measured by the Day-Night Stroop task, also made significant improvements in vocabulary skills over the prekindergarten and kindergarten years. None of the measures significantly predicted growth in emergent literacy development between prekindergarten and kindergarten.

**Table 5:** **Predictive validity: fixed effects model coefficients for growth in HTKS and other EF measures predicting growth in achievement across four waves (*N* = 205–207)**.

**Predictor**	**Mathematics^d^**			**Early literacy^e^**			**Vocabulary^f^**		
	***B***	***SE***	**β**	***B***	***SE***	**β**	***B***	***SE***	**β**
HTKS^a^	0.13	0.04	0.10^**^	0.00	0.09	0.00	−0.00	0.02	−0.00
DCCS^b^	0.36	0.11	0.09^**^	−0.11	0.22	−0.02	0.09	0.06	0.04
Day-Night Stroop	0.26	0.09	0.08^**^	−0.04	0.14	−0.01	0.10	0.05	0.06^*^
Working memory^c^	−0.04	0.04	−0.03	0.06	0.08	0.03	−0.00	0.01	−0.01
Simon Says	−0.28	0.36	−0.02	−0.24	0.61	−0.01	−0.06	0.20	−0.01

a The Head-Toes-Knees-Shoulders task.

b The Dimensional Change Card Sort task.

c The Woodcock-Johnson Auditory Working Memory subtest.

d The Woodcock-Johnson Applied Problems Subtest.

e The Woodcock-Johnson Letter-Word Identification subtest.

f The Woodcock-Johnson Picture Vocabulary Subtest.

* p < 0.05;

** p < 0.01.

## Discussion

Results demonstrated that in prekindergarten and kindergarten, children who scored higher on the HTKS also performed better on each of the individual measures of EF (cognitive flexibility, working memory, and inhibitory control) although the strength of these relations varied over time. In addition, REA indicated the HTKS and the EF measures significantly predicted variation in early achievement, with the strongest relations found for gains in early mathematics. In prekindergarten, measures of EF (especially the DCCS) were the strongest predictors of achievement in these models. In kindergarten, the HTKS was the most consistent predictor of achievement, although all measures of EF significantly predicted achievement depending on the time point. Results of the FEA found mostly consistent, albeit less strong, predictive relations compared to the random effects models.

### Construct validity of the HTKS

The current study sought to answer questions related to construct validity of a measure of behavioral self-regulation, called the HTKS. Previous research has differed on descriptions of what the HTKS measures, with some studies referring to the task as a measure of inhibitory control or response inhibition (Fuhs and Day, 2011; Lan et al., 2011), and some studies asserting evidence that it measures attention and working memory (McClelland et al., 2007a; Cameron Ponitz et al., 2009; Lan et al., 2011). Adding to this complexity, we have conceptualized it theoretically as a measure of behavioral self-regulation, to recognize the social context in which the HTKS is administered and demonstrates validity. This is consistent with a recent distinction of *EF* as a top-down cognitive process, that enables the *self-regulation* of a more automatic, bottom-up set of processes, such as one would demonstrate in a spontaneous social setting like a classroom (Ursache et al., 2012). Nonetheless, little research has examined the HTKS alongside traditional EF component measures. Furthermore, scholars of behavioral self-regulation and EF have been criticized for producing a plethora of “conceptual clutter” and “measurement mayhem” in the conceptualization and measurement of these skills (Morrison and Grammer, in press). If the construct of behavioral self-regulation is important for children's short- and long-term academic achievement, equally important is understanding how tasks like the HTKS are related to measures of EF, including assessments of cognitive flexibility, working memory, and inhibitory control.

We also found that children who performed better on the HTKS had better cognitive flexibility, working memory, and inhibitory control in prekindergarten and kindergarten, though the strength of associations changed over time. At early time points, the HTKS was most related to cognitive flexibility (the DCCS) and inhibitory control (Simon Says, Day-Night Stroop). In contrast, at later time points, the HTKS was most strongly related to the measure of working memory, although it was still significantly correlated with the other measures of EF. Correlations and regressions suggest that the HTKS shares significant variance with all measures of EF in prekindergarten and kindergarten. However, and of particular note, the strength of these relations also varies over time as demonstrated in the correlations and the regression results. It is possible that these developmental differences in the patterns of performance may relate to underlying developmental trajectories. For example, more specific EF components such as cognitive flexibility or inhibitory control may be important for less complex tasks, while tasks capturing multiple EF components like the HTKS may be more important for more complex tasks later in development. It appears that the HTKS may tap different aspects of EF at different points in early childhood, although those conclusions are also limited by the EF measures themselves and the analyses, which do not allow us to explicitly compare parameter estimates. It is difficult to find a pure measure of working memory, inhibitory control, or cognitive flexibility, especially in young children. This has been termed “task impurity” in the literature and reflects the overlap of many EF components in early childhood (Landis and Koch, 1977; Hughes and Graham, 2002; Best et al., 2009).

In light of these caveats, the results of the present study lend support to previous research arguing that the HTKS taps multiple aspects of EF, and extends this research by suggesting that inhibitory control may predominate in determining HTKS performance for younger children, attentional or cognitive flexibility is relevant from ages 4 to 6 years, and working memory may contribute more to performance for older children (McClelland et al., 2007a; Cameron Ponitz et al., 2009; McClelland and Cameron, 2012). The result showing that the HTKS was most strongly related to the measure of working memory by the end of kindergarten is conceptually consistent with the task demands as children progress through the task. The second and third parts of the task require that children remember a newly introduced set of rules (Part II) and then switch those rules (Part III). This is supported by preliminary evidence showing adequate variability in the HTKS, especially the third part of the task through age eight (von Suchodoletz, in preparation).

### Predictive validity of the HTKS and EF measures to academic outcomes

We also examined the predictive validity of the HTKS and measures of EF using REA, which model inter-individual differences in behavioral self-regulation and EF on academic achievement; and FEA, which model intra-individual change in a child's behavioral self-regulation or EF skills and intra-individual change in academic achievement. In contrast to previous research that questioned the unique role of EF in achievement (e.g., Willoughby et al., 2012b), present results supported the predictive validity of both the HTKS and measures of EF to growth in academic achievement using a variety of analytic strategies. Results of both REA and FEA in this study supported previous research that links behavioral self-regulation and EF with achievement over the transition to formal schooling. Consistent with previous similar research treating the child as a random effect, each of the measures that we tested significantly predicted children's academic achievement gains in prekindergarten and kindergarten. Within the random effects framework, this pattern indicates that initial levels of behavioral self-regulation, cognitive flexibility, working memory, and inhibitory control are each foundational for learning over time (Blair and Razza, 2007; McClelland et al., 2007a; Blair and Diamond, 2008). Scholars have argued that such skills enable children to make sense of and manage the multiple demands of classroom settings, and help create a set of habits that lead to continued successes (Diamond, 2010; Blair and Raver, 2012). Results indicated that some of the EF measures (especially the DCCS) were the strongest predictors of achievement during the prekindergarten year, whereas the HTKS was the most consistent predictor of achievement in kindergarten. It is possible that individual measures of EF may be most predictive of earlier achievement, while the relative predictability of a behavioral self-regulation task for later achievement increases as children get older and are faced with more complex demands.

The finding that each of the individual measures, which were moderately correlated, were associated with achievement growth may indicate that the behaviors children need to learn are somewhat diverse or, at least, can be captured with multiple measures. At the same time, domain specificity was observed where, in general, measures of behavioral self-regulation and EF showed their strongest and most consistent relations with mathematics and vocabulary, as compared with literacy. The HTKS was also the only measure to significantly predict gains in literacy skills. Theoretically, we have argued that behavioral self-regulation requires that children integrate all aspects of EF and perform in ways that are especially relevant for learning in school settings; this position could be empirically confirmed if an integrative measure like the HTKS were the best predictor of learning (McClelland and Cameron, 2012; McClelland et al., in press). The accumulating results for the HTKS using random effects models seem to support this position, but do not account for the fact that something else about the child, which both enables them to improve on the HTKS and to achieve academically over time, could explain the established links among the HTKS and later outcomes. Thus, we also examined our data using FEA.

Results of the FEA demonstrated similar, albeit less pronounced, patterns of predictability for the EF tasks and the HTKS measure of behavioral self-regulation. Measures of behavioral self-regulation (HTKS), cognitive flexibility (DCCS), and inhibitory control (Day-Night Stoop) significantly predicted growth in achievement between the fall of prekindergarten and the spring of kindergarten. The consistent significant finding for the HTKS and EF tasks and mathematics suggests that, during these early years, children who improved on measures of behavioral self-regulation and EF also demonstrated the most growth in mathematics. This finding matches a large body of evidence documenting strong links between children's EF and early mathematics (Blair and Razza, 2007; Bull and Lee, 2014). Reasons for this link can be tied to possible relations between specific components of EF and different aspects of early mathematics. For example, attentional shifting may be especially helpful for children to flexibly switch between multiple solutions to a math problem. In addition, inhibitory control may help children develop the types of learning-related behaviors that are needed to acquire early math skills, such as persistence and sequential problem-solving skills.

Our results suggest that aspects of EF and a measure of behavioral self-regulation are important for learning mathematics. Moreover, these results indicate that interventions to improve math might do well to target children's behavioral self-regulation as well as EF skills. Finally, children who made improvements on a measure of inhibitory control (the Day-Night Stroop task) also made significant gains in vocabulary skills between prekindergarten and kindergarten. Overall, this study, using two analytic methods, supports the robustness of the conclusion that behavioral self-regulation and EF component skills are important predictors of early academic achievement. However, in light of the reduced bias of unmeasured time-invariant variables, these results also suggest that the strength of prediction, although significant and substantial, may be somewhat lower than indicated by previous studies.

### Research and practical implications

At least two implications follow from the present study. First, the HTKS continues to demonstrate reliability and validity; and the measure seems to taps different aspects of EF although the strength of these relations varied over time between prekindergarten and the end of kindergarten. This is useful for researchers and practitioners who seek a short, economical, and psychometrically sound measure of behavioral self-regulation, which significantly predicts children's academic achievement—especially in mathematics—during the transition to formal schooling. Although researchers have emphasized the importance of using multiple measures of EF and behavioral self-regulation (Wiebe et al., 2008; Willoughby et al., 2012a), this may not always be feasible under time and budget constraints. The HTKS may be a practical alternative when it is not possible to use multiple measures and when predicting mathematics achievement is desirable (Duncan et al., 2007). Moreover, the minimal materials required for the task, coupled with its gross motor nature, make it an ecologically-appropriate measure for young children (McCabe et al., 2004).

The second implication is one for researchers, which points to continued examination of the constructs under investigation, but with the goals of parsimony, communication, and application. In early childhood, the dynamic development of multiple skill sets like EF and behavioral self-regulation means that, to some degree, we are studying a moving target. Furthermore, the use of distinct samples and measures introduces idiosyncrasies that contribute to the pattern of results for an individual study, yet are not well understood. It is one thing to draw conclusions about a construct from a single study, but researchers (including this author team) must also look across many studies to see the forest of EF components for the trees of what constructs and measures meaningfully predict whether or not children thrive in school. For example, the findings of this study may differ from those of Willoughby et al. (2012b) for multiple reasons, such as different measures or different sample characteristics.

It is also possible that relations between behavioral self-regulation and academic achievement may be reciprocal in young children. Recent research has demonstrated that an intervention focusing on academic skills in preschool led to significant improvements in academic outcomes and small improvements in EF (Weiland and Yoshikawa, 2013). Other research using cross-lagged models has found that the directionality is stronger from behavioral self-regulation to academic achievement than vice versa (Stipek et al., 2010), although more longitudinal work is needed. The overarching goal for scholars as well as teachers is not to increase scores on a behavioral self-regulation, EF, or achievement test *per se*, but to equip children with the general set of experiences and skills that will enable them to develop EF and demonstrate behavioral self-regulation within and beyond school settings (Blair and Raver, 2012). Furthermore, a number of interventions utilizing randomized controlled designs have demonstrated that interventions can significantly improve behavioral self-regulation and EF and academic achievement in young children (Bierman et al., 2008; Diamond and Lee, 2011; Raver et al., 2011; Tominey and McClelland, 2011; Schmitt et al., under review). Thus, despite continued refinement of terminology and methods, promoting behavioral self-regulation and EF in young children at home and at school is likely to help support their academic achievement and school success.

### Limitations

This investigation had some limitations. First, although the sample was socioeconomically diverse (50% low-income), it was less ethnically diverse with 61% of the children being White. This concern is somewhat ameliorated by previous research indicating that the HTKS is associated with achievement in diverse groups of children from different cultures Wanless et al., 2011a,b; McClelland and Wanless, 2012; von Suchodoletz et al., 2013; Wanless et al., 2013. In addition, the sample in the current study represented the demographic characteristics of the region in which it was drawn, but future research should include a greater diversity of children to better address this issue. Furthermore, covariates (i.e., Head Start status, parental education, and age) predicted attrition during year 1–2 of the study, and although these variables were used in the models with full information maximum likelihood to offset bias in estimates (Steiner et al., 2010), it is impossible to know if other unmeasured covariates were also related to attrition. Due to differential attrition and a non-random sample to begin with, generalizability of the findings might be limited and findings should be replicated in other studies. Second, it is possible that the presence of reduced variance (for instance, as seen in the Simon Says task at the fall of prekindergarten) could have limited the ability to detect significant associations between behavioral self-regulation and EF tasks and academic achievement outcomes. Third, although we used a variety of analytic strategies including FEA, we cannot infer causality from the results. As noted above, evidence from experimental studies indicate that improving children's behavioral self-regulation is likely to improve academic outcomes (Bierman et al., 2008; Diamond and Lee, 2011; Raver et al., 2011; Tominey and McClelland, 2011; Schmitt et al., under review), but more long-term research is needed. Finally, in the present study, all tasks were given to children by an assessor and not via computer. Thus, we were unable to measure information processing speed and use it as a control variable in our analyses. This is an avenue for future research.

## Conclusions

We examined the construct validity of a measure of behavioral self-regulation, the HTKS, assessing associations with measures of EF including cognitive flexibility, working memory, and inhibitory control. A second aim examined predictive validity of growth in the HTKS and EF tasks to academic achievement growth between prekindergarten and the end of kindergarten. Results indicated that the HTKS taps aspects of cognitive flexibility, working memory, and inhibitory control, although the strength of these relations varied between prekindergarten and kindergarten. In addition, the HTKS and EF tasks significantly predicted growth in academic achievement over 2 years in both random effects and fixed effects analyses (FEA). These results indicate that the HTKS, which takes 5–7 min to administer and does not require extensive materials, may be a practical tool that predicts children's achievement over the transition to kindergarten.

### Conflict of interest statement

The authors declare that the research was conducted in the absence of any commercial or financial relationships that could be construed as a potential conflict of interest.
